# Non-Olympic sports in a stratified sport system: perspectives on institutional constraints and sustainability

**DOI:** 10.3389/fspor.2026.1879308

**Published:** 2026-07-20

**Authors:** Simcha Avugos, Elia Morgulev, Yaniv Ashkenazi

**Affiliations:** 1 Levinsky-Wingate Academic Center – Wingate Campus, Netanya, Israel; 2 Kaye Academic College of Education, Beer-Sheva, Israel; 3 Ayelet – Israeli Federation of Non-Olympic Sport, Ramat HaSharon, Israel

**Keywords:** non-Olympic sports, public policy, resource allocation, sport governance, sport sustainability

## Abstract

Non-Olympic sports play important cultural, social, and community roles, yet their position within national sport policy frameworks remains under-examined. This study investigates the structural position of non-Olympic sports in Israel through the perspectives of coaches, many of whom also hold managerial responsibilities. Using a cross-sectional online survey, data were collected from coaches across diverse non-Olympic sports. Quantitative analyses assessed group differences by employment status and managerial role using descriptive statistics, one-way ANOVA, and eta squared (*η*^2^) effect sizes, while open-ended responses were analyzed using content analysis. Findings indicate that non-Olympic sports are perceived to occupy a peripheral position within a hierarchically organized sport system characterized by unequal access to funding, facilities, professional personnel, and media exposure, despite comparable levels of professional commitment. Drawing on sociological theories of capital, the results suggest that policy frameworks privileging Olympic performance concentrate economic and symbolic resources in recognized disciplines, reinforcing core-periphery dynamics. Although strong intrinsic motivation sustains engagement, responses to a hypothetical career-choice scenario warrant closer attention. By foregrounding governance structures and policy incentives, the study contributes to debates on stratification, sustainability, and access to resources and opportunities within sport systems, with implications extending beyond the studied context.

## 1 Introduction

The global sport system is heavily structured around the Olympic movement. Sports included in the Olympic program typically benefit from substantial media coverage, public recognition, and government support. In contrast, many sports operating outside the Olympic framework – often referred to as non-Olympic sports – receive considerably fewer resources and less media exposure. While this marginality is not universal across all non-Olympic sports, as illustrated by exceptions such as American football in the U.S., where high popularity and institutional strength coexist with non-Olympic status, it remains the case for most disciplines. These disparities reflect not only economic factors but also social hierarchies embedded within sport governance structures, which shape the perceived legitimacy and position of different sports.

Despite these structural differences, research on non-Olympic sports within the broader sport ecosystem remains scarce (1–3). Although sports such as rugby union, baseball, and cricket have been widely studied in single-sport contexts, their shared organizational and structural conditions have received considerably less attention. In particular, research has rarely examined how institutional arrangements, cultural valuation, and governance structures shape their development and sustainability as non-Olympic sports.

Non-Olympic sports encompass a wide range of disciplines, from traditional games such as cricket and rugby to niche sports like ultimate frisbee and roller derby, as well as emerging activities such as e-sports. This diversity reflects the fluid and evolving nature of what qualifies as a “sport”, particularly as digital competitions challenge traditional definitions centered on physical activity (4–6). Scholars have therefore noted an expansion in the conceptual boundaries of sport in response to cultural and technological shifts, increasingly including virtual and technology-driven forms of competition (7, 8). These shifts also carry sociological implications, as emerging sports reshape hierarchies of recognition within national and global sport systems. As new forms of competition continue to emerge, the distinction between traditional and non-traditional sports has become less clear, prompting researchers to reconsider how sports are defined, organized, and recognized within the global sporting landscape (9).

Beyond definitional debates, non-Olympic sports often play significant cultural and social roles. Many are closely tied to local identities and community traditions, serving as platforms for social cohesion and cultural expression (10–12). Examples include kabaddi in South Asia and hurling in Ireland, which are deeply embedded in local cultural traditions and community life (13, 14). In many regions, participation in such sports reflects historical traditions and shared cultural practices that extend beyond the competitive aspects of sport itself (15). At the same time, these sports frequently face structural challenges, particularly in securing funding, gaining media visibility, and establishing stable governance structures (16). The dominance of Olympic sports in the global sport industry further contributes to disparities in resources and public recognition (17). Consequently, many non-Olympic sports rely heavily on grassroots initiatives and volunteer-based structures to sustain participation and organizational activity.

Despite these constraints, non-Olympic sports are often associated with greater accessibility and inclusivity than Olympic sports, providing participation opportunities for individuals and communities that may be excluded from elite sport systems (18). Lower barriers to entry, flexible organizational structures, and community-oriented values often make these sports more open and welcoming to recreational participants and emerging athletes. Grassroots initiatives and participation in multi-sport events such as the World Games have also contributed to the gradual development and visibility of emerging sports, including korfball, dodgeball, and footgolf (19, 20). These pathways may serve as alternative routes for sports seeking greater legitimacy and international recognition outside the Olympic framework.

One particularly valuable perspective for understanding the structural and developmental challenges of non-Olympic sports is that of coaches. Coaches play a central role in athlete development and in the sustainability of sport programs. Examining their perspectives therefore provides insight into how these sports persist, adapt, and develop within a system dominated by Olympic sports, while also informing policy and management debates.

The international patterns described above are clearly reflected in the Israeli sport system, where Olympic and non-Olympic sports are institutionally separated and supported through different organizational frameworks. Sport in Israel is financed primarily through state budgets allocated via the Ministry of Culture and Sport and the Israel Sports Betting Council, as well as through municipal funding, commercial sponsorships, and sport centers. Public funding is distributed according to policy criteria that include an “Olympic coefficient”, which assigns greater weight to Olympic disciplines, contributing to disparities in resources and infrastructure (21). Consequently, non-Olympic sports operate under distinct organizational and financial constraints, making Israel a useful case for examining how governance and funding arrangements shape the experiences of coaches in these sports.

The present study focuses on a group of coaches in Israel, using their survey responses as key informants. The sample includes coaches with different employment statuses (primary vs. secondary occupation) and managerial roles (managers vs. non-managers). Olympic sports receive support through the Israeli Olympic Committee, whereas non-Olympic sports are overseen by the Ayelet Federation, which coordinates training, competitions, and community initiatives for sports outside the Olympic framework. These policy arrangements shape the everyday working conditions of coaches in non-Olympic sports.

This study makes three main contributions. First, it addresses a significant gap in sport management/governance literature by providing one of the few systematic empirical examinations of the structural positioning of non-Olympic sports – an area that has received limited scholarly attention beyond descriptive policy reports, both internationally and in Israel. Second, it advances understanding of how coaches – often overlooked stakeholders – perceive and navigate institutional inequalities within a dual sport system. Third, it offers a theoretically grounded account of how Olympic-centered policy frameworks reproduce and sustain hierarchies of legitimacy and resource allocation, thereby reinforcing core-periphery dynamics within contemporary sport systems. Although situated in Israel, the findings speak to broader sport systems in which Olympic/non-Olympic distinctions shape organizational opportunities and constraints.

## 2 Methods

### 2.1 Study design

The study employed a cross-sectional online survey design with an exploratory approach. Although it does not capture temporal change, the inclusion of active coaches provides a meaningful snapshot of the conditions under which non-Olympic sports operate.

### 2.2 Participants

The questionnaire was distributed to 89 coaches affiliated with the Ayelet Federation. Participants were recruited via a social media platform to which they belong. A total of 53 coaches completed the questionnaire (8 females) following repeated reminders. The sample represented a broad range of non-Olympic sports, including combat sports (karate, *n* = 13; muay thai, *n* = 5; kickboxing, *n* = 1; jiu-jitsu, *n* = 1; kendo-budo, *n* = 1), ball sports (American football, *n* = 3; cricket, *n* = 2; lacrosse, *n* = 1), movement and artistic sports (acrobatics, *n* = 4; dancesport, *n* = 2; roller sports, *n* = 4), precision and mind sports (pétanque, *n* = 4; lawn bowls, *n* = 1; bridge, *n* = 1), outdoor and adventure sports (orienteering, *n* = 2; skydiving, *n* = 2; water skiing, *n* = 2), and disc sports (flying disc, *n* = 4).

Twenty-three participants listed coaching as their primary occupation, while 30 held additional primary employment outside coaching. Thirty-four had managerial responsibilities at the club or national level in addition to their coaching role. Participants’ ages ranged from 17 to 73 years (M = 38.64, SD = 11.31), and coaching experience ranged from 1 to 31 years (M = 12.17, SD = 8.27). Nearly all coaches held some level of formal coaching accreditation, and 34 held an academic degree.

### 2.3 Instrument

The questionnaire was specifically developed for this study, based on relevant literature and established measures in coaching psychology and sport management (22–25). It was designed to capture four domains: (1) motivational drivers for coaching, including intrinsic and extrinsic factors; (2) perceptions of the relative position of non-Olympic sports compared with Olympic sports; (3) professional development, collaboration, and organizational support; and (4) professional preferences and perceived challenges.

The questionnaire underwent a process of face and content validity assessment through expert evaluation, following the procedure recommended by Hardesty and Bearden (26). A panel of four experts, all experienced coaches and managers in non-Olympic sports, evaluated the items for clarity and construct relevance. Their feedback informed revisions to the final version of the instrument.

The questionnaire was administered using Google Forms and consisted of 43 items. Items were rated on a 6-point Likert scale (1 = strongly disagree; 6 = strongly agree), with no neutral midpoint, in order to reduce central tendency bias (27). Four additional open-ended items allowed participants to elaborate on their experiences. Reliability for each section ranged from *α* = 0.85 to 0.90. The instrument was pilot-tested with a small group of coaches to ensure clarity and relevance of items.

### 2.4 Data analysis

Quantitative data were analyzed using descriptive statistics and one-way ANOVA to examine differences by occupation type (primary vs. non-primary) and managerial role. Statistical significance was set at *p* ≤ 0.05. Effect sizes were calculated to assess the magnitude of group differences and reported as eta squared (*η*^2^), with values of 0.01, 0.06, and 0.14 interpreted as small, medium, and large effects, respectively (28). Analyses were conducted using SPSS (version 20.0; IBM Corp., Armonk, NY, USA).

Open-ended responses were analyzed using conventional content analysis. Given the generally brief nature of the responses, an inductive coding approach was applied. Responses were first reviewed and then systematically coded by two independent coders and grouped into descriptive categories reflecting recurring patterns (e.g., motivations, perceived challenges). Discrepancies in coding were discussed until consensus was reached. These categories were used to complement and contextualize the quantitative findings, and selected quotations are presented to illustrate key patterns. The study was approved by the Institutional Review Board (IRB-00408), and confidentiality and anonymity were ensured for all respondents.

## 3 Results

Coaches whose primary occupation was coaching reported significantly more weekly hours than their colleagues with other primary jobs (M = 30.65 vs. 9.63; *p* < 0.001). No other significant differences were found for age, education, or coaching experience. Gender differences were not analyzed due to the small number of female coaches.

### 3.1 Motivation for coaching in non-Olympic sports

Economic incentives were generally low, including additional income (M = 2.19, SD = 1.42), attractive salary (M = 2.23, SD = 1.30), and good employment conditions (M = 2.47, SD = 1.35). Primary-occupation coaches rated salary and employment conditions significantly higher than non-primary occupation coaches (*p* = 0.001 and *p* = 0.006).

Idealistic motivations were rated highly across groups: improving the status of non-Olympic sports (M = 4.79, SD = 1.28), promoting an active lifestyle (M = 4.58, SD = 1.26), supporting others’ development (M = 4.81, SD = 1.19), and addressing professional staff shortages (M = 3.96, SD = 1.61), with no significant group differences.

Appreciation-related motivations were moderate. Coaches reported modest interest in proving themselves or receiving recognition from others (M = 3.13 and 2.83), with higher scores among primary-occupation coaches (*p* = 0.002 and *p* = 0.049). The overall valuation of the coaching profession was above moderate (M = 4.00, SD = 1.81), again higher among primary-occupation coaches (M = 4.65 vs. 3.50; *p* = 0.020).

All coaches reported high motivation for professional development (M = 4.89, SD = 1.33) and for exercising their personal skills (M = 4.91, SD = 1.33), with practicing the sport they love being the strongest motivator (M = 5.23, SD = 1.14). No significant differences were found based on managerial role, except for a near-significant difference in professional development scores among managers (M = 5.26 vs. 4.68, *p* = 0.123).

Significant differences by occupation type were associated with effect sizes ranging from medium to large (*η*^2^ = 0.074–.193), with the strongest effects observed for attractive salary, favorable employment conditions, and the desire to prove oneself to others, indicating that employment status was meaningfully related to several motivational dimensions.

 Table 1 summarizes the statistical results for occupation type (primary vs. non-primary) and the total sample.

**Table 1:** Perceived reasons for coaching in non-Olympic sports by employment status (primary vs. non-primary occupation).

Item	Non-primary M (SD)	Primary M (SD)	Total M (SD)	F	p	η^2^
Attractive salary	1.73 (1.14)	2.87 (1.22)	2.23 (1.30)	12.16	.001*	.193
Additional income	2.23 (1.36)	2.13 (1.52)	2.19 (1.42)	0.07	.796	.001
Good employment conditions	2.03 (1.13)	3.04 (1.43)	2.47 (1.35)	8.27	.006*	.140
Addressing professional staff shortages	4.17 (1.53)	3.70 (1.69)	3.96 (1.61)	1.12	.294	.022
Improving the status of non-Olympic sports	4.83 (1.26)	4.74 (1.32)	4.79 (1.28)	0.07	.793	.001
Promoting an active lifestyle	4.63 (1.19)	4.52 (1.38)	4.58 (1.26)	0.10	.753	.002
Contributing to others’ development and enjoyment	4.73 (1.39)	4.91 (0.90)	4.81 (1.19)	0.29	.592	.006
Exercising personal skills	4.67 (1.54)	5.22 (0.95)	4.91 (1.33)	2.27	.138	.043
Practicing a unique profession	3.50 (1.96)	4.65 (1.37)	4.00 (1.81)	5.77	.020*	.102
Pursuing professional development	4.70 (1.51)	5.13 (1.01)	4.89 (1.33)	1.38	.245	.026
Working in a sport I love	5.07 (1.31)	5.43 (0.84)	5.23 (1.14)	1.37	.247	.026
Proving myself to others	2.53 (1.61)	3.91 (1.47)	3.13 (1.69)	10.25	.002*	.167
Receiving recognition from others	2.47 (1.57)	3.30 (1.40)	2.83 (1.54)	4.08	.049*	.074

Note: (*) represents *p* ≤ 0.05.

Additional open-ended responses emphasized career growth, societal contribution, and personal passion. For example, coaches cited, “educating the next generation through sports” (Coach 12), “supporting athletes I believe in” (Coach 53), “I coach because of my love and passion for the sport” (Coach 27), and “I love this sport and it fills my life with energy and meaning” (Coach 29).

### 3.2 Perceptions of the position of non-Olympic sports

Coaches reported challenging conditions for non-Olympic sports, including insufficient budgets (M = 4.91, SD = 1.29), inadequate training facilities (M = 4.85, SD = 1.26), limited media coverage (M = 5.11, SD = 1.10), and lack of professional personnel (M = 4.42, SD = 1.39), as well as a generally poor public image (M = 4.94, SD = 1.01).

When directly compared with Olympic sports, non-Olympic sports were consistently seen as disadvantaged in resources and visibility. Both primary- and non-primary-occupation coaches perceived non-Olympic sports as having less funding (M = 5.22 vs. 4.90; *p* = 0.282), fewer training facilities (M = 5.00 vs. 4.67; *p* = 0.366), and lower facility quality (M = 4.26 vs. 4.23; *p* = 0.944) than Olympic sports. Coach 10 noted, “Suitable facilities and favorable training conditions are especially lacking in the more remote parts of the country.” Coach 15 added, “There isn't one single training facility that meets the required standards for coaching at an international level.” These perceptions were consistent across employment status groups, with no significant differences and effect sizes ranging from negligible to small (*η*^2^ = 0.000–.029).

Despite these constraints, coaches generally agreed that non-Olympic athletes (M = 2.75, SD = 1.66), coaches (M = 2.83, SD = 1.59), and managers (M = 3.40, SD = 1.55) are as professional as their Olympic counterparts, with no significant differences between primary- and non-primary-occupation coaches (*p* ≥ 0.223). As Coach 14 noted: “Olympic sports are overrated. They are no more interesting or challenging than non-Olympic sports. The only difference lies in the budgeting and the hype surrounding them.”

 Table 2 summarizes the statistical results for occupation type (primary vs. non-primary) and the total sample.

**Table 2:** Perceived structural conditions in non-Olympic sports by employment status (primary vs. non-primary occupation).

Item	Non-primary M (SD)	Primary M (SD)	Total M (SD)	F	p	η^2^
Lack of budgets	4.87 (1.33)	4.96 (1.26)	4.91 (1.29)	0.06	.804	.001
Lack of skilled personnel	4.53 (1.43)	4.26 (1.36)	4.42 (1.39)	0.49	.486	.010
Lack of suitable facilities	4.77 (1.22)	4.96 (1.33)	4.85 (1.26)	0.29	.592	.006
Lack of media coverage	5.00 (1.17)	5.26 (1.01)	5.11 (1.10)	0.72	.399	.014
Low public image	4.83 (1.18)	5.09 (0.73)	4.94 (1.01)	0.82	.369	.016
Fewer facilities than Olympic sports	4.67 (1.30)	5.00 (1.35)	4.81 (1.32)	0.83	.366	.016
Lower funding than Olympic sports	4.90 (1.13)	5.22 (0.95)	5.04 (1.06)	1.18	.282	.023
Less professional athletes than Olympic sports	3.00 (1.74)	2.43 (1.53)	2.75 (1.66)	1.52	.223	.029
Less professional coaches than Olympic sports	2.93 (1.51)	2.70 (1.72)	2.83 (1.59)	0.29	.595	.006
Lower management quality than Olympic sports	3.47 (1.63)	3.30 (1.46)	3.40 (1.55)	0.14	.709	.003
Lower facility quality than Olympic sports	4.23 (1.46)	4.26 (1.32)	4.25 (1.39)	0.01	.944	.000
Lower coaches’ salaries than Olympic sports	4.60 (1.48)	4.35 (1.30)	4.49 (1.40)	0.42	.520	.008

No significant differences were found between managers and non-managers in perceived managerial quality compared to Olympic sports. However, non-managers reported slightly higher agreement that facilities were inadequate (*p* = 0.053) and of lower quality (*p* = 0.074). Financial rewards were also perceived as lower than in Olympic sports, with slightly lower scores among primary-occupation coaches and coaches in managerial roles. Differences were not significant for either employment status or managerial role (*p* = 0.520 and *p* = 0.074, respectively).

### 3.3 Professional development, collaborations, and interactions

Support for professional development and guidance was relatively low (M = 3.26 and 2.92). Participation in development activities was moderate (M = 4.28, SD = 1.49), slightly higher among primary-occupation coaches (M = 4.57 vs. 4.07; *p* = 0.229). Non-managers reported higher participation than managers (M = 4.95 vs. 3.91; *p* = 0.013).

Preparation for the coaching role was rated moderately high (M = 4.52 vs. 3.97 for primary vs. non-primary; *p* = 0.131). Interactions with other coaches were mixed: consulting (M = 3.58, SD = 1.57) and observing sessions (M = 3.91, SD = 1.58) were low, whereas collaboration was higher (M = 4.17, SD = 1.53). Support and appreciation from affiliated sport associations (M = 3.30 and 3.15) and the Ayelet Federation (M = 2.89 and 2.94) were low, and sense of belonging was higher for sport associations than for the federation (M = 4.23 vs. 3.21). No significant differences were observed for either employment status or managerial role. Effect sizes across all items were negligible to small (*η*^2^ = 0.000–.044).

 Table 3 summarizes the statistical results for occupation type (primary vs. non-primary) and the total sample.

**Table 3:** Perceived professional development, collaboration, and organizational support by employment status (primary vs. non-primary occupation).

Item	Non-primary M (SD)	Primary M (SD)	Total M (SD)	F	p	η^2^
I have received appropriate training for my coaching role	3.97 (1.19)	4.52 (1.44)	4.21 (1.32)	2.36	.131	.044
I receive support for my professional development	3.13 (1.25)	3.43 (1.47)	3.26 (1.35)	0.65	.425	.013
I receive appropriate professional guidance	2.80 (1.32)	3.09 (1.38)	2.92 (1.34)	0.59	.446	.011
I participate in professional development activities	4.07 (1.74)	4.57 (1.04)	4.28 (1.49)	1.48	.229	.028
I consult with coaching colleagues	3.60 (1.77)	3.57 (1.31)	3.58 (1.57)	0.01	.937	.000
I collaborate with coaching colleagues	4.23 (1.68)	4.09 (1.35)	4.17 (1.53)	0.12	.733	.002
I observe other coaches’ training sessions	3.90 (1.73)	3.91 (1.41)	3.91 (1.58)	0.00	.977	.000
I receive support from my sport association in managing my team/athletes	3.43 (1.79)	3.13 (1.60)	3.30 (1.71)	0.41	.527	.008
I receive support from the Ayelet Federation in managing my team/athletes	3.07 (1.70)	2.65 (1.58)	2.89 (1.65)	0.82	.369	.016
I am appreciated by my sport association for my coaching achievements	3.20 (1.77)	3.09 (1.68)	3.15 (1.71)	0.06	.815	.001
I am appreciated by the Ayelet Federation for my coaching achievements	2.93 (1.82)	2.96 (1.64)	2.94 (1.73)	0.00	.962	.000
I feel a sense of belonging to my sport association	4.37 (1.50)	4.04 (1.69)	4.23 (1.58)	0.54	.465	.011
I feel a sense of belonging to the Ayelet Federation	3.37 (1.56)	3.00 (1.65)	3.21 (1.60)	0.68	.413	.013

### 3.4 Professional preferences and challenges

#### 3.4.1 Coaching preferences: non-Olympic vs. Olympic sports

Of 53 respondents, 22 preferred coaching in Olympic sports, 14 had no preference, 10 were undecided, and 7 specifically preferred non-Olympic sports. Olympic sports were valued for higher funding, better facilities, salaries, professionalism, institutional recognition, media coverage, access to talented athletes, and Olympic participation opportunities. Coach 10 explained:

“Budgets and access to facilities in Olympic sports are typically higher than in non-Olympic … If I coached an Olympic sport, I would be able to focus solely on the field that I love, instead of having to navigate between my two current jobs.”

Coach 19 similarly noted the prestige gap:

“The status of Olympic sports is much higher than that of non-Olympic sports, which struggle to achieve media coverage and face an acute lack of recognition.”

Coach 32 emphasized the competitive environment:

“Athletes at the high end are more motivated. They want to compete in the Olympic games, and that offers a more supportive environment for the athletes.”

Those preferring non-Olympic sports emphasized personal attachment to their discipline. Coach 7 said: “My sport isn't Olympic, but it's what I like to do, so that's why I do it.” Those with no preference highlighted general passion for coaching and national contribution.

#### 3.4.2 Professional challenges

Coaches reported multiple challenges, most frequently inadequate funding (*n* = 22) and insufficient facilities (*n* = 12). Other challenges included bureaucracy, limited federation support, difficulties in recruiting new talent, athlete dropout due to military service, lack of professional personnel, poor management practices, low sport popularity, insufficient financial rewards, equipment import difficulties, poor public image, and work-family balance.

Coach 37 described systematic shortcomings:

“There’s no systematic training framework for my specific sport … no proper management and planning … no follow-up training courses for coaches.”

He also noted the absence of athlete support services:

“There’s also no comprehensive support for the athletes such as physical therapy, nutrition, or mental training.”

Coach 13 highlighted everyday constraints:

“You always have to struggle for every additional training slot… clean the training facilities after sessions … request financial aid for travel expenses.”

Coach 42 emphasized financial limitations:

“Right now I'm actually coaching the national team for free. It's my donation.”

Finally, several respondents stressed broader institutional change, as Coach 6 stated:

“A fundamental shift is needed in the authorities’ attitude towards professional coaches. They should provide financial support instead of posing unnecessary obstacles.”

## 4 Discussion

### 4.1 General discussion

Sports governance in Israel operates within a regulatory framework anchored in national legislation, primarily the Sports Law (1988) (29), alongside governmental policy and public sport institutions. The law provides the legal foundation for sport associations, outlines general requirements for their operation, and establishes mechanisms for government support and oversight. The Ministry of Culture and Sport serves as the central governmental authority responsible for promoting and overseeing sport in the country. Within this mandate, it is involved in public funding allocation, facility development, and strategic policy planning. The law also requires that organized competitive sport operate through recognized national associations or federations, which regulate their respective sports in accordance with national frameworks and relevant international guidelines.

Against this broader governance context, the findings are interpreted as reflecting structural and organizational conditions in non-Olympic sport environments.

The findings indicate that non-Olympic sports in Israel operate under perceived structural disadvantages within the sport system, particularly in funding, facilities, media visibility, and institutional support. These patterns were supported by generally negligible-to-small effect sizes across analyses, indicating a consistent tendency of group differences. As these insights are drawn from individuals who often combine coaching and managerial responsibilities, they reflect the organizational realities of non-Olympic sport environments in the Israeli context. Such disparities align with broader research showing unequal resource allocation and institutional support across sports within national sports systems (23). Organizational environments and governance structures have long been recognized as key determinants of effectiveness within sport systems (22, 30), and the present findings suggest that these structural conditions may shape both the functioning and relative positioning of non-Olympic sports within the sport system.

More broadly, if sport is understood as a powerful social arena where cultural values, inequalities, identities, and political priorities are produced and contested (31), then the marginal position of non-Olympic sports may reflect not only managerial arrangements but also broader processes through which legitimacy and value are structured within the sport system. In this sense, the Israeli sport system appears to be hierarchically organized, where some sports are perceived as receiving greater economic and symbolic capital than others (32). The disadvantages described by the participants therefore reflect not only administrative gaps, but also broader perceived inequalities.

Despite these constraints, engagement within non-Olympic sports appears to rely heavily on intrinsic and value-driven motivations, such as promoting participation, fostering athlete development, and contributing to community life. Between-group differences in motivation, as reflected by effect sizes, were observed only in extrinsic and recognition-related factors. This is consistent with previous research emphasizing the role of personal commitment in sustaining involvement in resource-constrained sport contexts (24, 25). At the same time, the modest levels of appreciation received suggest that formal institutional mechanisms for acknowledging coaches’ contributions remain limited. From a management perspective, this may indicate an overreliance on individual commitment in the absence of formalized support and recognition structures. At a sociological level, such commitment can also be understood as a form of vocational orientation, where professional legitimacy derives from perceived social contribution rather than material reward [Weber's concept of *Beruf*, or “calling,” (33)]. However, when structural support remains weak, reliance on vocation and intrinsic motivation may lead to situations in which personal dedication compensates for institutional shortcomings.

The findings also suggest possible broader systemic implications of these structural disparities. Coaches’ preferences for Olympic sports were often associated with perceptions of greater funding, better facilities, and clearer professional opportunities. These findings align with sport policy research documenting how resource allocation models tend to prioritize Olympic sports with higher medal potential (23), thereby reinforcing existing hierarchies within the sport field. Such dynamics may be associated with differences in perceived career opportunities and could potentially be linked to a preference for better-resourced disciplines, although longitudinal evidence would be required to determine whether such preferences translate into actual mobility between sports. From a sociological perspective, these patterns may be interpreted as reflecting the interplay between agency and structure, whereby professional choices within the sport system appear shaped not only by individual preferences but also by structural conditions in the broader sport governance system.

At the same time, the results highlight the importance of organizational environments in supporting professional development within non-Olympic sports. While participants showed strong commitment to continuous learning, development efforts appeared largely self-directed, reflecting limited systemic support structures. Continuous professional development is widely recognized as a central component of effective coaching practice (34), yet research also emphasizes the role of supportive organizational contexts in enabling such development (30). In the present study, weaker affiliation with the umbrella organization (the Ayelet Federation) compared to sport-specific associations suggests that broader institutional frameworks may not fully address sector-wide needs. From a sociocultural perspective, professional learning is strengthened when practitioners see themselves as part of a shared community of practice (35). When ties between sports disciplines are limited, it may constrain joint capacity-building and weaken the ability of non-Olympic sports to articulate common interests within the wider sport system. Strengthening opportunities for mentorship, collaboration, and institutional recognition may therefore help address some of the organizational challenges identified here (36).

From a sport management and policy perspective, the findings suggest that improving the sustainability of non-Olympic sports requires attention to structural inequalities in funding, infrastructure, and professional opportunities. These disparities may not only affect athletes but also the working conditions, motivations, and career trajectories of coaches, who serve as key agents in sport development systems. While the study is situated in the Israeli context, similar resource allocation models and Olympic-centered policy frameworks exist in many national sport systems, suggesting broader relevance for policymakers (37). More broadly, the case illustrates how performance-oriented governance models shape hierarchies of legitimacy within contemporary sport systems, distinguishing between sports positioned at the symbolic “core” and those at the institutional “periphery”, regardless of actual athletic success. Addressing these gaps may therefore require more balanced funding criteria, investment in development pathways, and stronger institutional support structures for non-Olympic sports. Organizational initiatives such as structured professional development programs, mentorship networks, and formal recognition mechanisms could contribute to strengthening capacity, sustainability, and legitimacy across these sports.

### 4.2 Limitations and future research

This study is subject to several limitations. First, the findings are based on a single national context and self-reported perceptions of coaches, which may introduce subjectivity and limit broader generalization. Second, the questionnaire was specifically developed for this study and, although it underwent expert review and pilot testing, it was not subjected to full psychometric validation. Therefore, the findings should be interpreted in light of the exploratory nature of the instrument. Third, the sample focuses on coaches who also often hold managerial responsibilities, and therefore may not fully capture the perspectives of other stakeholders within non-Olympic sports.

Future research could extend this line of inquiry by adopting a comparative approach across sport systems with different governance structures and varying policy orientations toward Olympic and non-Olympic sports. Beyond cross-national comparison, further work may also examine how structural inequalities in recognition, legitimacy, and resource allocation are experienced by different social groups within the sport system, including athletes, administrators, and policymakers. Such research would enable a more comprehensive understanding of how sport governance reproduces or mitigates broader social hierarchies within contemporary sport systems. Longitudinal designs could further help capture how structural conditions and professional experiences evolve over time and shape professional trajectories and participation pathways in non-Olympic sports.

## 5 Conclusions

This study examined coaches’ perceptions of structural and professional conditions in non-Olympic sports. The findings indicate perceived constraints in resources and institutional support, alongside strong intrinsic and value-driven motivations for coaching engagement. Beyond these descriptive patterns, the main contribution of this study lies in offering empirical insight into non-Olympic sport contexts from the perspective of coaches as key agents within the sport system. It highlights how these less visible sport domains are experienced not only in terms of material conditions, but also in relation to professional standing, organizational positioning, and perceived legitimacy within the broader sport system.

For researchers, the study contributes to an expanded understanding of sport systems beyond dominant Olympic-oriented frameworks and highlights the need for greater attention to under-researched and structurally peripheral sport domains. For policymakers and sport organizations, the findings suggest that strengthening non-Olympic sports involves not only resource distribution, but also the development of institutional recognition, professional support structures, and clearer integration within national sport governance. Moreover, increasing visibility through more systematic media coverage and public communication strategies may help enhance awareness, legitimacy, and engagement in non-Olympic sports, thereby improving their position within the broader sport landscape.

## Data Availability

The original contributions presented in the study are included in the article/Supplementary Material, further inquiries can be directed to the corresponding author.
